# Supplementary material for: DNA-thioguanine nucleotide as a treatment marker in acute lymphoblastic leukemia patients with NUDT15 variant genotypes
Source: PLoS One. 2021 Jan 22;16(1):e0245667. doi: 10.1371/journal.pone.0245667 (PMC7822258; doi:10.1371/journal.pone.0245667)
Supplement: S1 Appendix — (DOCX) [file pone.0245667.s002.docx]

**S1 Appendix. Detailed description of study methods**

**The content of this supplementary index was referred to in the following article, and more details can be found in the mentioned article.**

Choi R, Chun MR, Park J, Lee JW, Ju HY, Cho HW, et al. Quantification of Thioguanine in DNA Using Liquid Chromatography-Tandem Mass Spectrometry for Routine Thiopurine Drug Monitoring in Patients with Pediatric Acute Lymphoblastic Leukemia. Ann Lab Med. 2021; 41:145-54. https://doi.org/10.3343/alm.2021.41.2.145 PMID: 33063676

*Methods for DNA-TGN measurement*

1. Materials

- Reagents; Sigma-Aldrich (St. Louis, MO, USA)
- TG, TG-d3, and guanine (G)-d3; Toronto Research Chemicals (Ontario, Canada)

1. DNA extraction

- MagNA Pure 96 system (Roche Diagnostics International Ltd., Rotkreuz, Switzerland)

1. Sample preparation for LC-MS/MS
   - Incubation of DNA (1 µg) in deionized water (75 μL) with derivatization buffer (1 M chloroacetaldehyde in 90 mM potassium phosphate; pH 5.0) (75 µL) at 99.9 °C for 3 h.
   - The sample was mixed with 0.2% formic acid (800 µL).
   - Conditioning and equilibration was done with 800 µL of methanol and 0.1% formic acid
   - The sample (1mL) was loaded onto a solid phase extraction (SPE) column (Strata X-C, 33 µm particle size, 30 mg/mL/well; Phenomenex, Torrance, CA, USA).
   - The adsorbed sample was washed with 800 µL of 0.1% formic acid, followed by 800 µL of 0.1% formic acid in 50% methanol and then eluted with 300 µL of 780 mM ammonium hydroxide in 50% methanol.
   - The eluate was dried at 40 °C under streaming nitrogen.
   - The dried eluate was reconstituted in 150 µL of 0.1% formic acid in 95% acetonitrile.
2. Calibrators
   - Calibrators were generated by spiking 10, 20, 100, 1,000, and 5,000 fmol TG into 1 µg pooled drug-free DNA from 20 volunteers.
     - Volunteers for DNA were note exposed to mercaptopurine.
   - Two samples for daily quality control (QC) were prepared (80 and 800 fmol TG/µg DNA using Jurkat cells).
   - An internal standard (IS) solution containing etheno-TG-d3 and etheno-G-d3 was prepared by derivatization of TG-d3 and G-d3 SPE.
   - A working IS solution with 1 µg/mL etheno-TG-d3 and 2 µg/mL etheno-G-d3 was prepared by diluting the stock IS in distilled water.
3. Chromatographic separation
   - Acquity UPLC System (Waters, Milford, MA, USA) coupled to a XEVO TQ-S tandem quadrupole mass spectrometer (Waters)
   - Ethylene-bridged hybrid hydrophilic interaction LC column (2.1×100 mm, 1.7 µm; Waters)
   - Injection volume; 5 µL
   - Total run time; 6 min/sample
   - Quantitative analysis was performed in the multiple reaction-monitoring mode with positive electrospray ionization (m/z 234.0→191.1 for etheno-TG, 237.0→194.1 for etheno-TG-d3, 176.1→94.1 for etheno-G, and 179.1→94.1 for etheno-G-d3).
   - Mass spectrometer settings
     - Source temperature, 150 °C; de-solvation temperature, 550 °C; capillary voltage, 3 kV; cone gas flow, 150 L/h; de-solvation gas flow, 800 L/h; and collision gas flow, 0.16 mL/min.

**Table 1.** Gradient conditions for chromatographic separation for DNA-TGN quantification

| Time segment | Time (min) | Flow rate (µL/min) | Mobile phase | |
| --- | --- | --- | --- | --- |
|  |  |  | %A* | %B† |
| 1 | Initial | 0.35 | 0 | 100 |
| 2 | 1.5 | 0.35 | 0 | 100 |
| 3 | 1.8 | 0.45 | 0 | 100 |
| 4 | 2.7 | 0.45 | 0 | 100 |
| 5 | 3.2 | 0.45 | 70 | 30 |
| 6 | 3.7 | 0.45 | 70 | 30 |
| 7 | 3.9 | 0.45 | 0 | 100 |
| *0.1% formic acid in distilled water; †160 mM formic acid and 10 mM ammonium formate in 95% acetonitrile. | | | | |

- - The MS response area was corrected with isotope-labeled TGd3 and G-d3.
  - Chromatographic etheno-TG peaks were normalized using etheno-G by calculating TG responses as etheno-TG area/etheno-G area (DNA-TGN=[etheno-TG response/etheno-G response]/[etheno-TG-d3 response/etheno-G-d3 response]).

*Methodology for validation of DNA-TGN quantification*

1. A total of five calibrators (10, 20, 100, 1,000, and 5,000 fmol TG/µg DNA) were used to generate daily calibration curves.

2. To determine intra-day (five independent analytical runs) and inter-day (five days) accuracy and precision, two QC samples (80 and 800 fmol TG/µg DNA) were used.

3. To determine the lower limit of quantification (LLOQ), control samples with 10 fmol TG were spiked into 1 µg DNA and tested in five replicated runs over three days.

4. The LLOQ; signal-to-noise ratio >10, coefficient of variation <20%, and bias <20%.

5. SPE recovery efficiency was evaluated in triplicate by spiking approximately 100 fmol etheno-TG into 1 µg of DNA in either the derivatized sample or from the SPE column eluate before LC-MS/MS quantification.

6. Extraction recovery (%) = area of derivatized sample/area of elute × 100

7. The effect of DNA on quantification was investigated by analyzing two linear calibration curves plotted for calibrators spiked into control DNA and distilled water.

8. Carry-over was investigated based on the response area of blank DNA samples measured immediately after measuring DNA samples containing 5,000 fmol TG/µg DNA. No significant carry-over was defined as the response area of the blank DNA sample being <20% of the LLOQ areas.

*Validated method specifications for LC-MS/MS of DNA-TG quantification*

1. Linearity

- 10-5,000 fmol TG/ug DNA
- -R^2^=0.9987 (R^2^ > 0.99)
- -y=0.0036x-0.0014

2. LLOQ

- 10 fmol TG/ug DNA
- S/N ratio 49 (S/N ratio > 10)
- bias -1.2% (bias < 20%)
- RSD 5.6% (CV < 20%)

3. Blank DNA (LOB)

- - Response area < LLOQ
  - < 20% of analyte and < 5% of internal standard

4. Extraction recovery (pre- and post- SPE response of e-TG)

- extraction recovery 85.7-116.2%
- Response area RSD 5.1-12.1% (RSD < 15%)

Abbreviations: TG, 6-thioguanine; LOB, limit of blank; LLOQ, lower limit of quantification; R2, coefficient of linearity; RSD, relative standard deviation; S/N, signal to noise; SPE, solid-phase extraction.
