# Supplementary material for: DNA-thioguanine nucleotide as a treatment marker in acute lymphoblastic leukemia patients with NUDT15 variant genotypes
Source: PLoS One. 2021 Jan 22;16(1):e0245667. doi: 10.1371/journal.pone.0245667 (PMC7822258; doi:10.1371/journal.pone.0245667)
Supplement: Attachment — Submitted filename: DNATGN_ResponseToReviewers_3rd_submit.docx [file pone.0245667.s004.docx]

**Response to the academic editor**

We sincerely appreciate the detailed review with suggestions. We have made corrections based on the comments. We sincerely hope that the manuscript is now suitable for publication in this journal and would be pleased to respond to any further queries regarding this submission. Thank you for your consideration.

1- Again, don't use "Will" throughout the text

🡪 Thank you for the detailed comment. We removed “will” from the text.

🡪 In addition to the few papers on DNA-TGN so far, this paper can (<- will) help establish the standard for therapeutic dose adjustment of 6MP based on DNA-TGN. (line 451)

🡪 In the future, larger studies are (<- will be) needed to reduce the 6MP dose in patients with *NUDT15* or *TPMT* variants and then titrate the dose based on DNA-TGN levels. (line 469)

2- Don't use "We, our". Use impersonal phrasing throughout the text

🡪 Thank you for the advice. We edited the sentences which included “we, our” as follows.

🡪 This study was able to demonstrate the efficacy of DNA-TGN in monitoring the treatment effects of 6MP, particularly in patients harboring *NUDT15* variant genotype. (line 481)

🡪 This appears to be the first study to analyze prospective serial DNA-TGN samples in a large number of patients—including those, which carry *NUDT15* variant genotypes—treated with reduced doses of 6MP. (line 455)

3- Revise Section heading to "Study strength and limitations" instead of the current one

🡪 Thank you for the advice. We changed the heading to “The significance of this study”.

4- Again, comments (8) 8- Line 177: Details regarding analysis, including method optimization and performance should be added as Suppl. Materials

🡪 Thank you for the advice which helps make the improved manuscript. We added the details regarding sample analysis, including method optimization and performance as supplementary material.

🡪 S1 Appendix. Detailed description of study methods.

5- Please check grammar and syntax errors with the assistance from native speaker

🡪 Thank you for the constructive advice. The manuscript underwent additional proofreading in English. English proofreading certificate is uploaded, also.
