# Supplementary material for: DNA-thioguanine nucleotide as a treatment marker in acute lymphoblastic leukemia patients with NUDT15 variant genotypes
Source: PLoS One. 2021 Jan 22;16(1):e0245667. doi: 10.1371/journal.pone.0245667 (PMC7822258; doi:10.1371/journal.pone.0245667)
Supplement: Attachment — Submitted filename: DNATGN_ResponseToReviewers_4th_submit.docx [file pone.0245667.s005.docx]

**Response to the academic editor**

We sincerely appreciate the detailed review with suggestions. Also, we apologize for some misunderstandings and improper corrections. We have made corrections based on the comments again. We sincerely hope that the manuscript is now suitable for publication in PLOS ONE. It will be of great help if a quick decision is possible. Thank you for your consideration.

==============================

ACADEMIC EDITOR: Again, authors have to amend the MS very carefully based upon the comments stated below

1- This is the 3rd time authors' are asked to revise the last paragraph before conclusion to "Study strength and limitations". Unfortunately, this is not done, although they have said done. Heading MUST be changed to what has been stated and related text should be in one paragraph

Response: We apologize for the misunderstanding and improper correction. We changed the heading to “Study strength and limitations” as recommended, and made the text into one paragraph.

2- Line 466-472: This is a redundancy for conclusion, avoid repetition

Response: We truly agree with your comment. As mentioned by the editor, we deleted the contents that overlap with the contents of the conclusion section.

3- Where is the results of method validation? Linearity, LOD, LOQ, Recovery, and RSD values. This should be added in the Suppl. Materials

Response: We do appreciate the detailed suggestion. We added the results of method validation for LC-MS/MS of DNA-TG quantification. In this study, LOB was experimentally confirmed, but LOD was not. (As two of the guidelines for LC-MS/MS method validation recommended LOD but was not recommended by other guidelines, LOD was not confirmed in this study.) Linearity, LOB, LLOQ, recovery, and RSD valued are presented.

4- Suppl. Materials should be checked as well for grammar and syntax errors

Response: Thank you for the comment. Supplementary index was checked for grammar and syntax errors, also.

5- All amendments should be highlights yellow. Don't use track-changes mode

Response: We appreciate for the advice. We highlighted the amendments instead of using track-changes mode.
